# Emergence and algorithmic information dynamics of systems and observers

**DOI:** 10.1098/rsta.2020.0429

**Published:** 2022-07-11

**Authors:** Felipe S. Abrahão, Hector Zenil

**Affiliations:** ^1^ National Laboratory for Scientific Computing (LNCC), 25651-075 Petropolis, Rio de Janeiro, Brazil; ^2^ Algorithmic Nature Group, LABORES for the Natural and Digital Sciences, 75005 Paris, France; ^3^ Oxford Immune Algorithmics, RG1 3EU Reading, UK; ^4^ The Alan Turing Institute, British Library 2QR, 96 Euston Rd, London NW1 2DB, UK; ^5^ Algorithmic Dynamics Lab, Unit of Computational Medicine, Department of Medicine Solna, Karolinska Institutet, 171 77 Stockholm, Sweden

**Keywords:** algorithmic information dynamics, emergence, observers, dynamical systems

## Abstract

One of the challenges of defining emergence is that one observer’s prior knowledge may cause a phenomenon to present itself as emergent that to another observer appears reducible. By formalizing the act of observing as mutual perturbations between dynamical systems, we demonstrate that the emergence of algorithmic information does depend on the observer’s formal knowledge, while being robust vis-a-vis other subjective factors, particularly: the choice of programming language and method of measurement; errors or distortions during the observation; and the informational cost of processing. This is called observer-dependent emergence (ODE). In addition, we demonstrate that the unbounded and rapid increase of emergent algorithmic information implies asymptotically observer-independent emergence (AOIE). Unlike ODE, AOIE is a type of emergence for which emergent phenomena will be considered emergent no matter what formal theory an observer might bring to bear. We demonstrate the existence of an evolutionary model that displays the diachronic variant of AOIE and a network model that displays the holistic variant of AOIE. Our results show that, restricted to the context of finite discrete deterministic dynamical systems, computable systems and irreducible information content measures, AOIE is the strongest form of emergence that formal theories can attain.

This article is part of the theme issue ‘Emergent phenomena in complex physical and socio-technical systems: from cells to societies’.

## 1. Introduction

The challenge of formalizing the notion of *emergence* usually centres on the definition of what the term ‘reducible’ (‘derivable’ or ‘predictable’) means when one says that a macro-level phenomenon is not reducible to its micro-level parts or to its initial conditions. In order to eliminate the possibility of one observer classifying a phenomenon as emergent while another classifies it as reducible to its isolated parts (to the parts at a smaller scale or to initial conditions), one approach is to define emergence as a property relative to the micro-level parts or to initial conditions [2–5]. The mathematical and empirical problem is to guarantee that such a dependence on the observer cannot occur even when formalizing emergence as a relative property [6]. In this article, to tackle this problem in the context of finite discrete deterministic dynamical systems (FDDDSs) (or computable systems in general), the act of observing is formally defined as an interaction in which the system being observed perturbs the observer while the observer perturbs the system being observed, where the observer is a particular type of system that can compute functions and is equipped with a formal theory. Hence, we show that mathematical measures of emergent behaviour as a relative property do depend on the formal theories that the observer brings to bear.

We show that despite being dependent on the observer’s formal knowledge, the emergence of algorithmic information is robust vis-a-vis variations of the arbitrarily chosen method of measuring irreducible information content, errors (or distortions) in the act of observing and variations of the algorithmic-informational cost of processing the information gathered from the observed system in accordance with the observer’s formal knowledge. In other words, all the subjective factors of language, measurement, information acquisition and processing are embedded into the definition of emergence of algorithmic information in such a way the formal theory (which the observer has brought to bear) is the only subjective characteristic that can determine whether or not the future behaviour of the observed system will appear emergent. This kind of emergence is called observer-dependent emergence (ODE).

Furthermore, we show that systems that display a sufficiently rapid increase of emergent algorithmic information overcome such dependence on the observer. In other words, there are systems whose behaviour eventually begins to display ODE for any observer. Although there may be an observer that can explain or predict a finite-length state space trajectory of an observed system, the sufficiently rapid increase of emergent algorithmic information guarantees that this will eventually cease to happen. In this case, the emergence of algorithmic information is guaranteed to be independent of any observer, but only at the asymptotic limit. This kind of emergence is called asymptotically observer-independent emergence (AOIE). The definition of AOIE inherits from ODE its robustness vis-a-vis the subjective factors of language, measurement, information acquisition and processing. However, one aspect of AOIE that is remarkable is that, unlike ODE, this is a type of emergence for which emergent phenomena will appear emergent no matter what formal theory that one might bring to bear.

We also compare these mathematical properties with previous models and definitions in the literature that deal with emergence in discrete deterministic dynamical systems and computable systems and with definitions of weak and strong emergence. We present an evolutionary model that displays the temporal (or diachronic) variant of AOIE and a model for networked systems that displays the holistic variant of AOIE. In particular, the latter model displays expected downward causation. Our results demonstrate that, restricted to the context of FDDDSs, computable systems and irreducible information content measures, AOIE is the *strongest* form of emergence that a formal theoretical approach can grasp. Further research is necessary for investigating whether or not the results in the present article can be extended to other physical, chemical or biological systems and other complexity measures.

In §2, we briefly present related work on emergence and information, focusing on the necessary distinction between stochastic and non-stochastic processes that delimits the context and conditions of our mathematical results. In addition, we introduce how the size of the algorithmic information content is quantified in FDDDSs or computable systems. In §3, we introduce the concepts of ODE and AOIE along with their underlying main ideas of observation and algorithmic perturbation. In addition, we present previous mathematical models in the literature that display ODE or AOIE. In §4, we compare these two kinds of emergence with definitions of weak and strong emergence. Finally, §5 concludes the article.

## 2. Emergence and information in stochastic and deterministic processes

For systems composed of (or defined by) stochastic processes, emergence has been studied in terms of statistical methods (for example, those based on entropy) and related complexity measures [7,8]. If an independent and identically distributed (i.i.d.) stochastic process $\left\{X_{i}\right\}$, where $X_{i}$ is a random variable, produces sequences of (finite) states, one knows from the noiseless source coding theorem that n H(X) gives a lower bound for the minimum expected number of bits to encode a sufficiently long sequence generated by this stochastic process [9], where H(X) is the entropy and n is the length of the sequence. In this context, due to such a minimality displayed by the entropy value in pure stochastic processes, the emergence of novel irreducible information can, for example, be understood as an entropy increase, as proposed in [7]. On the other hand, when emergence is interpreted as the appearance of a macro-level property that has greater efficiency of prediction than that of the micro-level states from which the macro-states derive, emergence has been proposed to be measured by employing a ratio between excess entropy and statistical complexity [8,10]. In the context of multivariate stochastic processes, causal emergence and downward causation have been proposed to be measured by employing variants of the unique information, which are based on the partial information decomposition [11] and integrated information decomposition [12].

However, in the context of deterministic processes, statistics faces insuperable obstacles when trying to quantify irreducible information content [13]. Being one of the well-known results in algorithmic information theory (AIT) [1], any resource-bounded computational procedure that tries to quantify the amount of irreducible information content in a single encoded object returns distorted values in general. Although the entropy of its contiguous blocks of length m is maximal, this distortion is, for example, seen in Borel-normal sequences of length n that are in fact computable (and therefore logarithmically compressible) [14], where m≪n. In the context of networks and graphs, there are also highly compressible graphs in which the degree-sequence entropy is maximal [15]. Thus, if one is interested in measuring irreducible information content (or measuring the emergence of new irreducible information) in deterministic systems, which are free of stochasticity, employing any fixed and computable measure based only on finding and exploiting statistical patterns (in order to approximate the most compressed form that computes the system’s behaviour) will exhibit limitations and face these obstacles in general. The main limitation stems from the fact that most computable patterns are not periodic, the kind of regularity that a statistical approach would be able to characterize. Computable but non-periodic patterns will tend to have high statistical complexity (e.g. Shannon entropy with no access to the underlying probability distribution) but low algorithmic complexity, meaning that a statistical approach would assign them a random nature that, for all mechanistic and cause-and-effect purposes, should not.

The present article only addresses discrete *deterministic* dynamical systems (or *computable* systems in general), and we quantify the irreducible information content of systems with algorithmic information content. As pointed out by Burgin [16], we refer to *algorithmic information content* of x as the minimum necessary and sufficient information for computably constructing x such that this information can always be extracted from x by a fixed function at some Turing degree. This way, the *size* ${\text{I}}_{\text{ac}}(x)$ *of the algorithmic information content* of x (see table 1 for a glossary of terms) is measured by the *equivalence class* of integer values $k\in {\text{I}}_{\text{ac}}(x)$ in the interval

2.1 $$|\text{K}(x)-k|\leq c_{\text{I}},$$

where $c_{\text{I}}\in N$ is an arbitrary and sufficiently large *object-independent* constant and K(x) is the (prefix) *algorithmic complexity* [17–20] (i.e. the length of the shortest prefix-free program $x^{*}$ that outputs the string x in a universal prefix Turing machine U). Note that this applies analogously to the size ${\text{I}}_{\text{ac}}(z\;|w)$ of the conditional algorithmic information content of z given w, which is an equivalence class of values $k\in {\text{I}}_{\text{ac}}(z\;|w)$ in the interval

2.2 $$|\text{K}(z\;|w)-k|\leq c_{\text{I}},$$

where the *conditional* prefix algorithmic complexity of a binary string z given a binary string w, denoted by K(z |w), is the length of the shortest program $z_{w}^{*}$ such that $\text{U}(\left\langle w,z_{w}^{*}\right\rangle )=z$ and ⟨⋅,⋅⟩ denotes the arbitrarily chosen encoding of the pair (x,y), where x,y∈N. Note that ⟨⋅,⋅⟩ can be recursively extended to ⟨⋅,⋯,⋅⟩ in order to represent the encoding of n-tuples. The constant $c_{\text{I}}$ may depend on the choice of the observer (but not on the object) and it can be taken as sufficiently large as possible so as to ensure that the constant-bounded equivalence class ${\text{I}}_{\text{ac}}(\cdot )$ becomes *invariant* and *minimal* with respect to a particular observer [1].

**Table 1.** Table of mathematical notation and acronyms

${\text{I}}_{\text{ac}}(x)$	the size of the algorithmic information content of an encoded object x	Section 2
${\text{I}}_{\text{ac}}(y|x)$	the size of the (conditional) algorithmic information content of an encoded object y given an encoded object x	Section 2
FDDDS	acronym for finite discrete deterministic dynamical system	Section 2
$c_{\text{ I }}$	constant that determines the equivalence class ${\text{I}}_{\text{ac}}(\cdot )$	Section 2
$S_{t}$	a single state of a FDDDS S at time instant t	Section 2
$S{↾}_{t}^{t^{\prime }}$	a state space trajectory $\left(S_{t},\ldots ,S_{t^{\prime }}\right)$ of S from the state $S_{t}$ until state $S_{t^{\prime }}$	Section 2
P	an algorithmic perturbation	Section 3
O	an observer Turing machine, which is a particular type of two-tape Turing machine whose first tape receives input and the second tape stores formal knowledge	([1], Section 3.2)
O	a formal observer system, which is a particular type of FDDDS that simulates O	Section 3
$S_{t}^{\prime }$	a single state of S at time instant t that occurs after S being perturbed/observed at any time instant <t	Section 3
$c_{O}$	constant that determines the minimum conditions for the observation to take place	Section 3
ODE	acronym for observer-dependent emergence	Section 3a
$c_{e}$	constant that determines the minimum conditions for observer-dependent emergence	Section 3a
AOIE	acronym for asymptotically observer-independent emergence	Section 3b
P	a sequence (or collection) of Turing machines	Section 3b(i)
S	a discrete deterministic dynamical system whose each contiguous subsequence of its entire state space trajectory is a state space trajectory of a particular FDDDS $S^{\left(k\right)}$	Section 3b(ii)
S	a macro-level discrete deterministic dynamical system whose micro-level systems (or parts) *cannot* interact with each other	Section 3b(ii)
$S^{\prime }$	a macro-level discrete deterministic dynamical system whose micro-level systems (or parts) *do* interact with each other	Section 3b(ii)

When dealing with other kinds of objects that are not strings, a mathematical object is said to be *encoded* if it is univocally represented by structured data so that there is an algorithm which can always recover or extract the original object from the structured data. This way, one can equivalently define ${\text{I}}_{\text{ac}}(x)$ and ${\text{I}}_{\text{ac}}(z\;|w)$ when x, w and z are encoded objects instead of strings.

Moving to the context of discrete dynamical systems, let $S=(X_{S},f_{S},E_{S},T)$ be a FDDDS embedded in an environment E [5], where $X_{S}$ is the state space of S,

2.3 $$\begin{array}{lcccc} f_{S} & : & X_{S}\times E_{S}\times T & \to & X_{S}\\ & & \left(S_{t},e_{S_{t}},t\right) & \mapsto & S_{t+1} \end{array}$$

is the function that defines the *evolution rule* of S, $E_{S}$ is the space of all possible surrounding environmental states that constitute the boundary of S and T is the set of time instants. If the cardinality of the set $E_{S}$ of a dynamical system S is finite, then the dynamical system is said to have a finite *boundary*. If both sets $X_{S}$ and $E_{S}$ are composed only of discrete finite states, the finite-boundary dynamical system is said to be *finite* and *discrete*. The environment $E=(X_{E},r_{E},T)$ is a FDDDS into which the systems S and their environmental surroundings $E_{S}$ are embedded, where $r_{E}:X_{E}\times T\to X_{E}$ is the evolution rule of E. In case the evolution rule of a dynamical system is a computable function, or computable relation, then the dynamical system is said to be *computable*.

We define the measure of the *size of the algorithmic information content of a FDDDS* $S=(X_{S},f_{S},E_{S},T)$ from time instant t until time instant $t^{\prime }$ by ${\text{I}}_{\text{ac}}(S{↾}_{t}^{t^{\prime }})$, where $S{↾}_{t}^{t^{\prime }}$ is just a notation for an arbitrary encoding of the sequence $\left(S_{t},S_{1},\ldots ,S_{t^{\prime }}\right)$ of states (i.e. *a state space trajectory* of S from t∈T until $t^{\prime }\in T$).

It is known that Turing machines can be simulated by computable FDDDSs. For example, one can construct an *elementary cellular automaton* employing Rule 110 that simulates a Turing machine [21]. Moreover, the decision problem of one is reducible to the decision problem of the other and the time complexity of the Turing machine simulation by elementary cellular automata can be improved to a polynomial time overhead [22].

Furthermore, in instances where the system S is simulating an arbitrary Turing machine w and the decision problem of S until t is *Turing equivalent* to the decision problem of U(w), one can equivalently measure the algorithmic information content of S by ${\text{I}}_{\text{ac}}(y)$ instead of ${\text{I}}_{\text{ac}}(S{↾}_{0}^{t})$, where U(w)=y ([1], Lemma 2.1). And the conditional case ${\text{I}}_{\text{ac}}(\cdot |\cdot )$ applies analogously.

Algorithmic information-based approximation methods to the size of the irreducible information content are proved to be accurate in the asymptotic limit when the computational resources are unbounded. In addition, due to the property of always existing ‘room for improvement’ in resource-bounded compression algorithms, empirical applications of the theoretical results presented in this article are agnostic with respect to the chosen compression algorithm. These properties hold because: (i) since for any arbitrarily chosen encoding method or universal prefix-free programming language, its value can only vary by a constant that does not depend on the object, algorithmic complexity is an *invariant* measure of irreducible information content [17–20]; (ii) it also is *minimal* because, for any arbitrarily chosen formal method of assigning a probability distribution μ(⋅) to the infinite discrete space of computably constructible objects, the value −log⁡(μ(x)) can only be smaller than the algorithmic complexity K(x) up to an object-independent constant [19,20], where x is an encoded object; and (iii) the exact value of K(x) is not computable in general, but there are always new algorithms that are able to produce better approximations than previous algorithms. Thus, although perceiving the behaviour of a particular system as emergent depends on the observer’s formal knowledge, one of the important contributions of this article is that the values of algorithmic information content remain invariant and minimal with respect to this observer [1].

Future research is necessary for investigating in which conditions the theoretical results in the present article can be extended to stochastic processes and other complexity measures.

## 3. Dependence on the observer in emergent phenomena

The introduction of perturbation (or intervention) analysis, in the context of algorithmic information content, enables the investigation of the underlying computable causal effectiveness of its parts (or elements) [23,24] and offers a solution to the inverse problem of finding the best generative model [25]. A generative model in the context of algorithmic information means a step-by-step computable model (which in turn means being able to be carried out by a Turing machine) that generates the object, data sample or system to be analysed. Such an introduction of perturbation analysis led to the introduction of algorithmic information dynamics (AID) [26], based on the (expected) universal optimality of algorithmic probability [1] and stems from the demonstrated high convergence rate of computable generative models to their algorithmic probability [13]. This is a rate stable to radical changes to the model of computation, that produces a stable distribution in particular for low complexity and thus high algorithmic frequency (probability) objects. (Note that the ultimate convergence is guaranteed by the invariance theorem [17–20].) We show in this article that one of the paradigm shifts brought about by AID vis-a-vis previous methods based on computability and information theory is that perturbation analysis guarantees that our results hold, even if we allow the very act of observing to substantially change (or introduce ‘noise’ into) the observed system’s behaviour.

In order to formally capture the notion of what is an observation in the context of FDDDS, or computable systems in general, one needs to define what one means by an algorithmic perturbation of the states of a system and specify the distinctive characteristics of a FDDDS that renders the latter as being an observer.

Since any state space trajectory of a FDDDS is a sequence of finite discrete states, then, for every perturbation of a state $S_{t}$ at time instant t that results in the next state $S_{t+1}^{\prime }$, there is at least one computer program (or Turing machine) that performs this exact change by taking $S_{t}$ as input and outputting $S_{t+1}^{\prime }$. Intuitively, an algorithmic perturbation occurring at a certain time instant changes the course of the state space trajectory from that moment on. This way, an algorithmic perturbation P is defined as such a program that changes the course of the state space trajectory of a FDDDS by taking the previous (not yet perturbed) state $S_{t}$ of this FDDDS as input and outputting a future state $S_{t+1}^{\prime }$, which is distinct from what the next state $S_{t+1}$ of the FDDDS would have been if *no* perturbation had occurred. In other words, an algorithmic perturbation occurring at time instant t is any kind of external algorithmic process that updates the state of the affected system after one time step, resulting in a new state at time instant t+1. That is, instead of the state $S_{t+1}$ that S should display if no perturbation had occurred, S displays $S_{t+1}^{\prime }$ after an algorithmic perturbation has occurred at time instant t. More formally, an *algorithmic perturbation* P at time instant t is a perturbation occurring at the time instant t of a state space trajectory $\left(\ldots ,S_{t}\right)$ of a FDDDS S that updates the one time step from t to t+1 so that, instead of the original state space trajectory $\left(\ldots ,S_{t},S_{t+1},\ldots \right)$, it results in a distinct state space trajectory $\left(\ldots ,S_{t},S_{t+1}^{\prime },\ldots \right)$, where P is a program and $\text{U}(\left\langle S_{t},P\right\rangle )=S_{t+1}^{\prime }$.

So, as a more concrete illustrative example, suppose every state of a FDDDS S is a 3-bit string and suppose $S_{t}=001$ and $S_{t+1}=010$. If a perturbation occurs at time instant t and it leads the next state to be $S_{t+1}^{\prime }=011$ instead of $S_{t+1}=010$, we know that there is at least one algorithm that corresponds to this exact perturbation. Such an algorithm can be as simple as ‘read the first two bits of the input and flip the second bit, then returns the resulting 3-bit string’, where $P_{2}$ is a program of a Turing machine that represents this algorithm so that $\text{U}(\left\langle 001,P_{2}\right\rangle )=011$.

Note that the existence of an algorithmic perturbation does not depend on where it comes from or on the nature of the process that caused the perturbation. What mathematically follows from the definition of algorithmic perturbation is simply that: any finite state change in a FDDDS S can be reduced to, or represented by, an equivalent algorithmic perturbation into S; and that any halting program P on input $S_{t}$ is a possible algorithmic perturbation that may (or may not) occur on system S at time instant t. Whether or not one is assigning probabilities to the occurrence of perturbations depends on the problem and model to be studied. From the example in the previous paragraph, suppose one knows beforehand that the second bit was flipped due to a stochastically random event (with probability 1/3) in which the bit to be flipped is selected randomly. But, since the result of such a perturbation just produces 011 out of 001, then that change can be equivalently represented by the algorithmic perturbation $P_{2}$ or any other algorithmic perturbation P such that U(⟨001,P⟩)=011. Thus, if $P_{2}$ is the algorithmic perturbation one chooses (possibly, because $|P_{2}|$ is minimal) to represent the stochastically random event of flipping the second bit, which is a stochastic perturbation that occurs with probability 1/3, then the probability of occurrence of the algorithmic perturbation $P_{2}$ also becomes 1/3.

While the conditional algorithmic complexity of any perturbation that results in 011 from the past state 001 is constant and very small—because the simplest algorithmic perturbation P for which U(⟨001,P⟩)=011 holds can only be as complex as flipping the second bit—finding the algorithmic complexity of the equivalent algorithmic perturbations to stochastically random perturbations on more complex objects, such as networks, is less trivial. In the case of monoplex networks (or graphs), it is shown in [26,27] that stochastic randomly deleting (or inserting) |F| edges in a network G, which results in a new network $G^{\prime }$, is equivalent to applying an algorithmic perturbation $P_{F}$ to G such that

3.1 $$\text{K}(P_{F})\leq 2|F|\log_{2}(N)+\text{O}(\log_{2}(|F|))+\text{O}(\log_{2}(\log_{2}(N))),$$

and $\text{U}(\left\langle G,P_{F}\right\rangle )=G^{\prime }$, where F is the subset of edges that were perturbed and N is the number of vertices. This is because the right side of the inequality in equation (3.1) is an upper bound for the conditional algorithmic complexity of the shortest program that, with the network G as input, can perform the same edge deletions (or edge insertions) that the stochastically random deletion (or insertion) of |F| edges did.

For example, suppose the probability that a destructive stochastic perturbation deletes a *single* edge is $\frac{2}{N^{2}-N}$. We know there is an equivalent algorithmic perturbation $P_{F_{1}}$ such that $\text{K}(P_{F_{1}})\leq 2\log_{2}(N)+\text{O}(\log_{2}(\log_{2}(N)))$ [26,27]. If one chooses $P_{F_{1}}$ to represent that exact stochastically random deletion, then the probability of occurrence of $P_{F_{1}}$ will also be $\frac{2}{N^{2}-N}$. Indeed, as one of the important properties implied by this equivalence in AID, a stochastically random perturbation on a single edge can only change the final algorithmic complexity of the network by $\text{O}(\log_{2}(N))$ bits, which explains the thermodynamic-like behaviour found in [24] about the reprogrammability of networks when these are subjected to stochastically random single-edge perturbations. More specifically, this thermodynamic-like phenomenon refers to a larger number of one-by-one stochastically random edge deletions (or insertions) being necessary for transforming an algorithmically random (i.e. incompressible) network into a low-algorithmic-complexity network than the number necessary for transforming a low-algorithmic-complexity network into an algorithmically random network. This is because (stochastically) random single-edge deletion on algorithmically simple networks has a greater impact than (stochastically) random single-edge deletion on algorithmically random networks [24]. On the one hand, to lower the algorithmic randomness of an algorithmically random graph, *non*-stochastic single-edge deletion is required. On the other hand, to turn a low algorithmic complexity network into a higher algorithmic complexity network, stochastically random single-edge deletion suffices [27].

A *formal observer system* O is a particular type of FDDDS that has prior knowledge of a formal theory and can simulate Turing machines (or compute functions) during its state space trajectory. Thus, as demonstrated in [1, Section 3.2], whether or not a formal observer system can compute a certain function is a fact dependent on the prior formal knowledge that the formal observer system knows. This is an important property that we will explore in §3a. A direct consequence of the definitions of algorithmic perturbation and formal observer system is that they enable an algorithmic perturbation to change the machine that O is simulating ([1], Lemma 3.1). For example, suppose $\left(O_{0},\ldots ,O_{t}\right)$ is a state space trajectory that simulates a two-tape Turing machine that computes the value of $f(w_{1})$ when $w_{1}$ is encoded as input in its first tape and the second tape contains an encoding of a formal theory F, where f is a total computable function. Then, suppose that an algorithmic perturbation occurs at time instant t, giving rise to the next state $O_{t+1}^{\prime }$ instead of the original state $O_{t+1}$, which was supposed to occur if no algorithmic perturbation had happened at time instant t, so that $O_{t+1}^{\prime }$ is the initial state of the FDDDS that simulates the same two-tape Turing machine of O but with the first tape containing $w_{2}$. So, note that the FDDDS O after the algorithmic perturbation has occurred at time instant t will be simulating the two-tape Turing machine that computes the value of $f(w_{2})$ when $w_{2}$ is encoded in its first tape. Therefore, $\left(O_{0},\ldots ,O_{t}\right)$ computes the value of $f(w_{1})$ and $\left(O_{t+1}^{\prime },O_{t+2}^{\prime },\ldots \right)$ computes the value of $f(w_{2})$.

In general, the observation of an object by the observer should be realized when the interaction between them somehow sends sufficient information about the observed object to the observer. In case both the observer and the observed object are systems, this interaction becomes understood as a mutual perturbation in which the system (the observer) perturbs an object (another system), while being itself perturbed by the object. This way, the observation of a system by another system is realized when such a mutual perturbation results in a sufficient amount of information obtained by the observer during the observation so that it informs about the behaviour of the system being observed. In summary, the act of observing is a particular type of mutual perturbation in which sufficient mutual information is preserved between the system being observed and the knowledge obtained by the observer during the act of observing. In the context of FDDDSs and algorithmic perturbations, the act of observing occurs when both the formal observer system and the observed FDDDS are algorithmically perturbing each other in such a way that the post-perturbation behaviour of the formal observer system contains sufficient *algorithmic information* about the behaviour of the observed FDDDSs. This is formalized in [1, Definition 3.4].

As demonstrated in [1], this definition of the act of observing is general enough to encompass the case in which observation takes place, but it is defective. That is, when O observes S at time instant t and it only obtains partial information about S. In other words, this defective information about S can differ from the actual information about S, but only up to a bounded error margin (given by the constant $c_{O}$ that does not depend on S and only depends on O), which instantiates and delimits the subjective nature of the act of observing. Intuitively, this observation may only be acquiring partial information, and not all the desired information, due to: either intrinsic limitations of the properties of the formal observer system, such as limited sensory capabilities or measurement accuracy; a stronger effect of the algorithmic perturbation $P_{\left(O,S,t\right)}$ from O into S at time instant t; or both. In all such examples, the subjective character of the formal observer system is evinced, subjectivity which is reflected in the value of the constant $c_{O}$.

In addition to defective observations, in some cases the observation can be *ideal* or *perfect*. An ideal observation takes place not only when the constant $c_{O}$ is small, but also when there is a fixed program p (which does not depend on both the observer and the observed system) that computes the behaviour of the observed FDDDS. Thus, a perfect observation is understood to be perfect or ideal not only because the particular observer gathered all the information about the observed system’s behaviour so that there is an algorithm that can retrieve the very observed system’s behaviour from the internal states of the observer, but also because this holds from the perspective of any possible observer that knows that algorithm. Changing the scope to stochastic processes instead of deterministic processes, this notion of perfect observation may be tightly connected to the concept of perfect observation of a (stochastically) random variable in [28]. Polani [28] defines a perfect observation as one where the number of random variables that constitute the observer is sufficiently large so that the conditional entropy of the observed system, given these random variables, is as small as one wishes. Then, the increase of intrinsic information within all these variables over time is proposed as a measure of self-organization. Further research is necessary to investigate how the results of the present article are related to emergence and dependence on the observer in the context of stochastic processes.

### (a) ODE

Intuitively, emergence of algorithmic information occurs when the formal theory known by the observer is not sufficient for computing, predicting or completely explaining the system’s future behaviour from its constituent parts or prior conditions. In the process of trying to explain or predict the behaviour of an observed system, the observer employs the resources available, its own previously held formal knowledge and the information it could gather from the observation.

The main idea of ODE is that, even if one takes into account equivalent methods to measure the irreducible information content (given the presence of the constant $c_{\text{I}}$), the error margin of defective information in the observation (given the presence of the constant $c_{O}$), and the algorithmic informational cost of processing all the information that the observer could gather (given the presence of a constant $c_{e}$ which also only depends on the observer and not on the observed system), there is still an insufficient amount of algorithmic information to compute the future behaviour of the observed system ([1], Definition 4.1). That is, all the subjective factors of measurement, information acquisition and processing considered, the formal observer system still cannot produce a sufficient amount of algorithmic information in order to be able to compute the future state space trajectory of the observed FDDDS. More specifically, the presence of the three object-independent constants $c_{\text{I}}$, $c_{O}$ and $c_{e}$ sets the extent to which such an invariance and robustness of ODE hold when the formal observer system is trying to compute or predict the behaviour of a system. Because these constants depend on the formal observer system and not on the object (i.e. the observed system), they serve the dual purpose of expressing the capabilities of the formal observer system, while still taking into account the inherent subjectivity of the formal observer system, which is the distinctive feature of ODE [1]. In summary, the comprehensiveness of ODE is that, after these three subjective constants are set to values as large as one wishes (but still finite), the only subjective characteristic of the formal observer system that can change whether the future behaviour of the observed FDDDS appears emergent or not is the formal theory held by the observer.

As demonstrated in [1], the future behaviour of the observed FDDDS may appear emergent to a formal observer system, while non-emergent to another formal observer system. This is because, if a finite *extra* amount of algorithmic information is sufficient for the first formal observer system to predict the emergent behaviour of the observed FDDDS, then this finite extra amount of algorithmic information can always be converted into a new extended version of the formal theory, which the first observer had. Hence, the second formal observer system equipped with this new extended formal theory can compute the behaviour of the observed FDDDS that was considered to be emergent by the first observer. To the second observer, the behaviour of the observed FDDDS ceases to appear emergent. Thus, in cases which the emergence of algorithmic information results from a lack of a *finite* amount of algorithmic information, these emergence phenomena can be classified as being dependent on the observer precisely because of this dependence on the prior formal knowledge.

As an illustrative example of this dependence, suppose that Peano arithmetics is consistent (i.e. it does not prove contradictions) and that a formal observer system simulates a two-tape Turing machine whose first tape is empty while its second tape contains an encoding of the axioms of Peano arithmetics, so that this two-tape Turing machine tries to prove whether or not the received input in the first tape is a true arithmetical sentence by only using Peano arithmetics. Suppose now that an algorithmic perturbation during the observation causes the arithmetical sentence ‘Con(PA)’ (which asserts the consistency of Peano arithmetics) to be encoded into the first tape of such a formal observer system. Then, we know that this two-tape Turing machine will never halt, and therefore the formal observer system will necessarily be simulating a *non-halting* program. This holds because of the incompleteness of Peano arithmetics [29].

Now, suppose that another formal observer system simulates a two-tape Turing machine whose first tape is empty and has the axioms of Peano arithmetics plus the extra axiom ‘Con(PA)’ encoded into its second tape. Then, in case an algorithmic perturbation during the observation causes the arithmetical sentence ‘Con(PA)’ to be encoded into the first tape of such a formal observer system, the formal observer system will be simulating a *halting* program that proves the consistency of Peano arithmetics.

In [4], a weakly emergent phenomenon is defined as one for which the macro-level states of a system can only be derived by simulating the system itself. Later, Bedau [30] refines the notion of derivability in this definition, introducing the notion of explanatory incompressibility. For example, in Conway’s Game of Life, one cannot in general decide from the initial configurations whether or not a macrostate behaviour will have a certain property. Only by simulating the game would it be possible to gain sufficient irreducible information about whether or not the macrostate behaviour has a specific property.

Bedau [4,30] characterizes weak emergence in an informal approach that does not specify the role of the observer, how the micro-level states are observed, how one decides whether or not something is derivable or incompressible and the time instants when the events occur. Nevertheless, for present purposes, one can assume a free interpretation of the notions presented in [4,30] and translate the definition of weak emergence into the context of FDDDSs, algorithmic perturbations and algorithmic information as a slight variation of ODE ([1], Definition 4.1) by adding to the incompressibility condition the condition of the existence of another observer that can compute the state space trajectory by simulating the very state space trajectory [1]. This way, since such a ‘simulation irreducibility via explanatory incompressibility’ in [4,30] implies that there are conditions in which [1, Definition 4.1] is satisfied, and vice versa, we argue that the weak emergence described in [4,30] can be understood as an informal alternative, but one that is *equivalent* to the ODE ([1], Section 4.1.1).

It is claimed in [4] that the simulation irreducibility is a property that is not dependent on the current limited knowledge of the observer. Under our interpretation of the ‘simulation irreducibility via explanatory incompressibility’, this claim becomes true or false depending on whether or not one restricts the possibilities of the formal theory that any formal observer system may have access to. For example, if every observer (which does not simulate the observed system) can only know the same formal theory and all of them are subjected to the same constants $c_{e}$, $c_{O}$ and $c_{\text{I}}$, then one can argue that such a claim as made in [4] indeed holds. However, in case any formal theory may be encoded into the second tape of a formal observer system, one can straightforwardly employ ([1], Definition 4.1) to demonstrate that such a claim becomes false (see [1], Section 4.1.1).

Another model for FDDDSs in which a type of emergence occurs may be found in [3,31]. If the interaction of a (finite) dynamical system A with its environment E (i.e. another finite dynamical system) gives rise to a recurrent state space trajectory whose length is greater than the length of all the other recurrent state space trajectories of any isolated system of the same size as A, the pair of systems A and E is said to exhibit *unbounded evolution*. If the newly emerging recurrent state space trajectory from the interaction between A and E is *not* contained in any of the other recurrent state space trajectories of any isolated system of the same size as A, the pair of systems A and E is said to exhibit *innovation*. In addition, since interaction with the environment can introduce state-dependent changes in the evolution rules of system A, the increase of the recurrence time shown in the models investigated in [3] can be classified as an example of emergence through *downward* (or *top-down*) *causation* [3,32,33]. Downward causation is usually described in the literature as a type of process in which the global (or macro-level) dynamics of the system as a ‘whole’ gains causal efficacy over the micro-level systems (or parts) [6,32,34].

Note that in the models of cellular automata in [3], the interaction with the environment can produce changes, i.e. perturbations, in the evolution rule of system A. So it differs from algorithmic perturbations because the latter impact the states of system A instead of its evolution rule. In fact, one can reduce each state-dependent rule perturbation of a cellular automaton A in [3] to an equivalent algorithmic perturbation on an equivalent universal cellular automaton that emulates A. To this end, just note that every finite cellular automaton is computable by a Turing machine and there are universal cellular automata (for example, elementary cellular automaton Rule 110) that can simulate any Turing machine [1]. Thus, for each state-dependent rule perturbation in [3], one can construct such an equivalent algorithmic perturbation and then demonstrate that there is a constant $c_{e}$ for which ODE (as in [1], Definition 4.1) implies unbounded evolution and innovation (see [1], Section 4.1.1). Additionally, it can be employed to prove that unbounded evolution and innovation define a type of emergence that is dependent on the observer’s prior knowledge ([1], Section 4.1.1). Thus, the kind of emergence from unbounded evolution and innovation is dependent on the observer’s formal knowledge, and one can always choose a constant $c_{e}$ such that unbounded evolution and innovation is implied by the ODE. In attempting to show that the two approaches are equivalent, it is important to note that models displaying an empirical tendency toward an increase in algorithmic complexity were investigated in [3]. In this line of research, the inverse problem (that is, to prove that a FDDDS displaying unbounded evolution and innovation *always* implies that there is at least one constant $c_{e}$ for which the state space trajectory of [1,Definition 4.1] is satisfied) constitutes necessary theoretical research that remains to be done.

### (b) AOIE

The next question that naturally arises is whether such an approach can be extended to formalize an emergent phenomenon that continues to be emergent for any observer.

The definition of AOIE also imports from ODE the robustness vis-a-vis the subjective factors of measurement, information acquisition and processing. However, the distinctive main defining idea is that a FDDDS displays AOIE if, for every formal observer system, there is a certain stage of the FDDDS from which a certain yet unavailable amount of algorithmic information begins to be necessary to compute the future behaviour of the observed FDDDS (see [1], Definition 4.2). In other words, for every formal observer system, there is a certain stage at which the subsequent behaviour of the observed system begins to display the ODE. There might be a formal observer system that can compute a finite-length state space trajectory of the FDDDS, but if it displays AOIE, then it is guaranteed that this formal observer system will eventually cease to be able to compute its behaviour. This is the reason AOIE is an emergence that is guaranteed to be *independent* of the observer, but only at the *asymptotic limit*. For example, at each stage of a FDDDS displaying AOIE, the strongest emergence that it can display to a particular formal observer system is ODE, but eventually any other formal observer system that might try to compute the subsequent behaviour of the FDDDS will be outdone and, therefore, any other formal observer system will eventually also experience ODE.

Emergent phenomena can usually be divided into two kinds [6,35]: a *temporal* version in which emergence occurs over time as the system interacts with the environment (for this reason it is called *diachronic* emergence [6,34]); and a *holistic* (or synchronic) version in which emergence occurs as a distinctive feature of the ‘whole’ in comparison to the parts [6,34]. Suppose a system displays the temporal variant of the AOIE. In this case, for every observer, there is a time instant that triggers a phase transition for which, if the behaviour of the system does not appear emergent to the observer until such time instant, then it will start to appear emergent after this time instant. Now, suppose another system displays the holistic variant of the AOIE. In this case, for every observer, there is a distinctive macro-level characteristic (e.g. the size of the system or the number of constituent parts) that triggers a phase transition for which, if the behaviour of the system does not appear emergent to the observer until such a characteristic is manifested, then it will start to appear emergent once this characteristic manifests. In the following §3b(i),(ii), we will first present an evolutionary model that displays the temporal AOIE. Then, we will present a model for networked systems that display the holistic AOIE.

#### (i) A model and example of temporal AOIE in evolutionary systems

In [5,36,37], a theoretical model for the open-ended evolution of programs (or Turing machines) is presented with the purpose of obtaining a mathematical proof of Darwinian evolution within the framework of AIT. Inspired by (but not limited to) evolutionary biology, this field is called *metabiology* and in a general sense it constitutes a pursuit of mathematical proofs of meta-level fundamental properties and quintessential ‘laws’ in evolutionary systems [38].

The cumulative evolution model is defined in [36,37] as a sequence of sole (resource-unbounded) Turing machines that evolve over time due to the transformations effected by randomly generated algorithmic mutations. Thus, in accordance with evolutionary biology, not only are these Turing machines subjected to randomly generated mutations and natural selection, but they may also inherit information from their predecessors. Chaitin [36] presents a theoretical analysis of the expected sufficient number of algorithmic mutations for reaching a fitness value that necessarily requires n bits of irreducible information content to be computed. Indeed, in the cumulative evolution model, n bits of algorithmic information is proved to be achieved over a realistically small number of randomly generated mutations. Due to the known mathematical properties of algorithmic information such as invariance and minimality, this shows that a quantity of irreducible information content is achieved in a realistically fast mutation time through randomly generated mutations applied to evolving programs that can inherit past information. In particular, Chaitin [36] demonstrates that n bits of algorithmic complexity is expected to be reached after $\text{ O }(n^{2}{\left(\log(n)\right)}^{2})$ randomly generated algorithmic mutations. This result is achieved by employing a theoretical analysis of the resulting algorithmic complexity from certain algorithmic mutations that are expected to occur over time. Abrahão [39,40] then demonstrated that the results for resource-*unbounded* Turing machines in the former cumulative evolution model trickle down to the realistic resource-*bounded* case: n bits of *time-bounded* algorithmic complexity is expected to be reached after $\text{ O }(n^{2}{\left(\log(n)\right)}^{2})$ randomly generated *time-bounded* algorithmic mutations in the cumulative evolution of *time-bounded* Turing machines.

These abstract evolutionary models were then corroborated by empirical results in [41], not only showing that randomly generated algorithmic mutations produce a speed-up in adaptation in comparison with the uniformly random point mutations (which are the usual random mutations under consideration in mainstream models based on evolutionary modern synthesis), but also that it may be related to explanations of the occurrence of modularity, diversity explosions and massive extinctions.

We note that algorithmic mutations as in [36,37] are exactly the algorithmic perturbations we introduced in §3, except that in these particular evolutionary models, the algorithmic perturbations are randomly generated by following the usual i.i.d. probability distribution of prefix-free binary sequences.

In this manner, one can combine the result from Chaitin [36] and the definition of AOIE in order to demonstrate the existence of an evolutionary process that displays AOIE ([1], Theorem 4.1). The main idea is that the cumulative evolution of sole Turing machines under successive perturbations performed by the randomly generated algorithmic mutations—an evolutionary process which results in an infinite sequence P of Turing machines—is able to guarantee (with a probability as high as one wishes) that the emergence of algorithmic information in the resulting sequence P is larger than any formal observer system can keep up with in the long run.

The kind of emergence shown in such an evolutionary AOIE falls under the diachronic variant of AOIE. In particular, the *open-endedness* proved in [36] strictly refers to the unbounded increase of complexity over time, as the evolution unfolds. For this reason, it is called *evolutionary open-endedness* [2,42]. Another example of diachronic emergence and open-endedness is the one presented in [3], which we discussed in §3a. As we have already demonstrated, the distinctive feature of emergence in the diachronic AOIE is that it is asymptotically independent of the observer’s formal knowledge, while emergence in [3] is dependent on the observer’s formal knowledge. In this sense, we can also adopt the convention of classifying evolutionary AOIE as *asymptotically observer-independent diachronic open-endedness* and the emergence in [3] as *observer-dependent diachronic open-endedness*.

#### (ii) A model and example of holistic AOIE in networked systems

The pervasiveness of non-homogeneous network topological properties has fostered the recent field of network science and showed its important role in complex systems science [43]. In this regard, motivated by the pursuit of a unified theory of complexity in network science and complex systems science [44,45], the theory of *algorithmic networks* [2,42,46] allows the investigation of how network topological properties can trigger emergent behaviour that is capable of irreducibly increasing the computational power of the whole network. An algorithmic (complex) network N is a population of computable systems whose members can share information with each other according to a complex network topology. Each node of the network is a computable system and each (multi-dimensional) edge of the network is a communication channel.

In [2], it is shown that there are network topological properties, such as a small diameter, associated with a computationally cheap and simple communication protocol of plain diffusion that asymptotically trigger an unlimited increase in expected emergent algorithmic complexity of a networked node’s final output as the number of nodes increases indefinitely. The diameter of a network (or graph) is the length of the longest shortest path from any node to any other node [47]. The diameter is said to be small if the diameter grows by a logarithmic order of the number of nodes [43] and such a small-diameter phenomenon is one of the important properties found in both real-world and synthetic networks [43,48].

The unlimited increase of expected emergent algorithmic complexity obtained in [2] is called expected emergent open-endedness (EEOE) [2,42] and—by simplifying the notation from Abrahão *et al.* [2,42] to serve our present purposes—it may be mathematically defined by

3.2 $$\lim_{N\to \infty }{\text{E}}_{N}(\Delta_{iso}^{net}\text{ K }(o_{i},c))=\infty ,$$

where: ${\text{E}}_{N}(\cdot )$ gives the average value over all possible randomly generated nodes in the algorithmic network N; $\Delta_{iso}^{net}\text{ K }(o_{i},c)$ is the *emergent algorithmic complexity* of a node $o_{i}$ in c communication rounds, which is defined by the difference between the algorithmic complexity of the node $o_{i}$ in c communication rounds when running *networked* and the algorithmic complexity of the node $o_{i}$ in c communication rounds when running *isolated* from any other node; and N is the total number of nodes in the algorithmic network N. Note that the limit in the definition of EEOE eventually neutralizes any pair of constants $c_{\text{ I }}$ that one may subtract or add to this difference. Thus, one can equivalently define the EEOE as

$$\lim_{N\to \infty }{\text{E}}_{N}(\Delta_{iso}^{net}{\text{I}}_{\text{ac }}(o_{i},c))=\infty .$$

In case an algorithmic network displays EEOE, this means that the expected algorithmic information necessary to explain the networked behaviour of a node eventually starts to grow faster than the expected algorithmic information necessary to explain the isolated behaviour of the same node. That is, as the number of nodes grows toward infinity, the algorithmic complexity of the networked behaviour of the node is increasingly larger, on average, than the algorithmic complexity of the isolated behaviour of the node.

The proof of the occurrence of EEOE in the models studied in [2] is achieved by employing a theoretical analysis of the trade-off between the number of communication rounds and the average density of networked nodes with the maximum algorithmic complexity. There is an optimum balance between these two quantities where, if a large enough average density of these nodes is achieved in a sufficiently small number of communication rounds, then EEOE is triggered.

Instead of the communication protocol of plain diffusion employed in [2], Abrahão *et al.* [42] show that a susceptible-infected-susceptible contagion scheme [49,50] in algorithmic networks with a power-law degree distribution is also sufficient for triggering EEOE. In [51], it is shown that a slight modification in the communication protocol of plain diffusion from Abrahão *et al.* [2] is sufficient for enabling the whole algorithmic network to synergistically solve problems in a higher computational class than the computational class of its individual nodes. These results show that network topological properties found in complex networks can indeed trigger emergence of increasing computational power of the whole network with respect to its constituent parts.

As we mentioned in §3b(i), it was shown in [39,40] that the evolutionary open-endedness from Chaitin [36,37] also applies to the resource-bounded case. Regarding the EEOE from Abrahão *et al.* [2,42], further research is needed for establishing how a resource-bounded version of the EEOE in algorithmic networks unfolds mathematically.

Let $\text{S}=(S^{\left(1\right)},S^{\left(2\right)},\ldots ,S^{\left(k\right)},\ldots )$ be a FDDDS in which every contiguous subsequence of its entire state space trajectory is a state space trajectory of a particular $S^{\left(k\right)}$ (see [1], Section 4.2 for a formal definition). Now, to show the existence of the holistic variant of AOIE, we slightly extend our notation to encompass the case of *macro-level* dynamical systems that are composed of other *micro-level* dynamical systems. This will allow us to further explore the necessity of extra algorithmic information at a certain stage of a system when the macro-level dynamical system has reached a sufficiently large size. Let S denote a FDDDS from which each (macro-level) state $S{↾}_{t}^{t}$ at time instant t is a fixed arrangement of all the (micro-level) states ${\text{S}}_{i}{↾}_{t}^{t}$ with 1≤i≤N, where t is an arbitrary time instant and N is the total number of dynamical systems in the form ${\text{S}}_{i}$ that composes S. So, each FDDDS ${\text{S}}_{i}$ is a constituent part of the larger FDDDS S and the organized collection of all ${\text{S}}_{i}$, with 1≤i≤N, defines the entire S ([1], Section 4.2.2). For example, in case S is a bidimensional FDDDS, each state of S at time instant t can be represented as a matrix in which each entry represents a single state of ${\text{S}}_{i}$ at time instant t, where N=mn and m is the number of rows and n is the number of columns. The choice of the arrangement of the micro-level FDDDSs is arbitrary and, as long as this choice is fixed, it does not change our final results.

Now, we construct the FDDDS ${\text{S}}_{i}$ until time instant t, corresponding to the node $o_{i}$ running isolated. Secondly, we construct the FDDDS ${\text{S}}_{i}^{\prime }$ from time instant t+1 until $t^{\prime }$, corresponding to the node $o_{i}$ running networked. Thus, a node $o_{i}$ receiving information from its neighbour nodes (according to the network topology) is equivalent to the FDDDS ${\text{S}}_{i}^{\prime }$ being algorithmically perturbed by its neighbour FDDDSs (according to the same network topology). Then, we combine these micro-level FDDDSs ${\text{S}}_{i}$ and ${\text{S}}_{i}^{\prime }$ in order to form the macro-level dynamical systems S and $S^{\prime }$, which refer to the isolated and networked case, respectively. Summing up, $S^{\prime }$ is a population of randomly generated FDDDSs that can perturb each other according to the network topology. On the other hand, although S is composed of the same population of $S^{\prime }$, no FDDDS in S can perturb other FDDDSs in S.

In this way, one can show that the state space trajectory of any of the micro-level systems ${\text{S}}_{i}^{\prime }$ of the macro-level $S^{\prime }$ is expected to display AOIE with a probability as high as one may wish as the network/population size increases indefinitely ([1], Theorem 4.2). That is, even if a formal observer system can computably predict the expected behaviour of an isolated micro-level system ${\text{S}}_{i}$, there is a phase transition for which, if the expected behaviour of a networked micro-level system ${\text{S}}_{i}^{\prime }$ does not appear emergent to this observer, then it will start to appear emergent once the number of micro-level systems is sufficiently large.

While the temporal (or diachronic) variant of AOIE presented in the previous §3b(i) occurs over time due to successive perturbations from the environment into the system P, the variant of AOIE in the previous paragraph occurs due to the interaction (in the form of perturbations) between the micro-level systems ${\text{S}}_{i}^{\prime }$ as the number of these micro-level systems (contained in the macro-level system $S^{\prime }$) increases. Thus, the sort of process that gives rise to such an AOIE differs from the one in §3b(i) in the same manner as the holistic variant of emergence differs from the temporal (or diachronic) one. For this reason, AOIE in the previous paragraph falls under the *holistic* variant of emergence. Hence, one can adopt the convention of calling this kind of *open-endedness* in the holistic variant of AOIE *asymptotically observer-independent holistic open-endedness*.

The holistic variant of AOIE demonstrates that, for sufficiently large $S^{\prime }$, the expected behaviour of a networked micro-level system $S_{i}^{\prime }$ is overruled by the algorithmic-informational dynamics of the algorithmic perturbations produced according to the network topology. This occurs because the algorithmic information of the dynamics of an isolated ${\text{S}}_{i}$ is eventually not sufficient for computing the expected networked behaviour of ${\text{S}}_{i}^{\prime }$, while the total algorithmic information shared through the network is. Thus, it constitutes an *expected downward causation* in FDDDSs (or in networked computable systems), which also offers the advantage of this expected downward causation being independent of the observer at the asymptotic limit.

## 4. Weak, intermediate or strong emergence

In a broad sense, if weak emergence is characterized by phenomena that are *in principle* deducible or derivable from the simple initial or micro-level conditions, but that appear as unexpected at a higher coarse-grained level due to the lack of information, resources or knowledge, one can classify the ODE in §3a as weak emergence. This agrees with the approach to weak emergence as unexpectedly complex behaviour in [6], as explanatory incompressibility in [30] (see §3a), and as type 0 and 1 weak emergence in [35]. Indeed, since there is always the possibility of another observer existing to which the phenomenon ceases to appear emergent, then ODE is, ‘*in principle*’, deducible or derivable at the same time that there are observers for which the emergent behaviour is ‘truly’ incompressible and relatively uncomputable. The term ‘truly’ is employed here in the precise sense that such an incompressibility or relative uncomputability does not depend on the method chosen to measure the information content, on the errors or distortions in the act of observing itself, or on the algorithmic-informational cost to process the information gathered from the observed system in accordance with the observer’s formal knowledge.

On the other hand, classifying the AOIE in §3b is not so easy. The crux of the matter lies not quite in the notion of reducibility, derivability or predictability (as in our case they have a formal unambiguous translation into sufficient algorithmic information) as in the phrase ‘in principle’. If ‘in principle’ means that the phenomenon should remain emergent for every formal observer system that belongs to the same computational class as the observed systems, then AOIE could be interpreted as a type of strong emergence. This is because for every formal observer system of the same computational class (e.g. with the same Turing degree or in the same running time complexity class) of the observed state space trajectories, the behaviour of an observed system that displays AOIE will eventually cease to be computable or predictable in the long run.

For example, assume a Church–Turing hypothesis in which everything our mathematical theories can infer or formalize by employing step-by-step effective methods is attainable by finitely axiomatizable theories, or by Turing machines. As a consequence, our mathematical theories will not be able to, in general, decide whether or not the infinite asymptotic behaviour of a state space trajectory of a FDDDS continues to be uncomputable by those theories. At the same time, the information carried or conveyed by any finite-length state space trajectory of a FDDDS can always be added to prior formal theories in order to construct more overarching new formal theories. Therefore, since AOIE implies ODE for infinitely many time steps in the future, the Church–Turing hypothesis entails that a system displaying AOIE (and, in this case, strong emergence) will always be understood as displaying ODE (and therefore the aforementioned weak emergence), while in fact never ceasing to display ODE (or weak emergence) for any possible mathematical theory we might devise. In other words, under the Church–Turing hypothesis, if AOIE is considered strong emergence, then this type of strongly emergent phenomenon is a pseudoparadoxical type of emergent phenomenon that is always mathematically understood as weak emergence by us, while in fact displaying strong emergence (if an hypothetical observer could know the point of view of every observer). Note that, not only under the Church–Turing hypothesis, such a pseudoparadox of emergence also holds as long as every observer and every observed state space trajectory of a system belong to the same computational class.

Another form of strong emergence has been described as the ultimate necessity of novel fundamental powers or laws to scientifically explain the macro-level behaviour of a system [34]. In the context of FDDDSs or computable systems, AOIE offers a proof of this necessity, but now formally expressed as the never-ending necessity of new axioms (or new algorithmic information).

Type 2 strong emergence is introduced in [35] as a type of emergence that occurs when the behaviour of a macro-level system is sufficiently influenced by constraints (or external factors) to which the macro-level system is subjected, and these constraints do not apply to (or cannot be derived from) the micro-level constituent parts. Thus, due to the presence of expected downward causation in §3b(ii), one can also successfully argue that the systems $S^{\prime }$ satisfy type 2 strong emergence in [35].

However, if ‘in principle’ does not restrict the computational class of the observer, then AOIE can be brought back to the weak case. This is because although no (finite) formal axiomatic theory held by the observer can compute the observed system in the long run, there may be *oracle* observers that can, if the evolution rule of the observer itself belongs to higher Turing degrees. For example, it is true that both the sequence P of Turing machines in §3b(i) and the macro-level FDDDS $S^{\prime }$ in §3b(ii) cannot be computed by formal observer systems at the asymptotic limit, but both can be computed by an oracle machine of Turing degree  0' . In other words, if one allows observers to have access to an infinite source of algorithmic information, e.g. by filling out the *infinite* second tape of O with a halting probability (or Chaitin’s Omega number) [17,52], there are systems that satisfy the definition of AOIE at the same time that they are relatively computable by a *special* observer. Thus, in cases where one believes in the existence of strong emergence that resists ontological characterization in terms of physical or informational causal efficacy, such as the emergence of qualia in the conscious mind [6,34], it becomes consistent to classify AOIE as a type of weak emergence.

Although not constraining the computational class of the observer may seen reasonable, one is inherently assuming that there are ‘special’ observers that belong to a higher computational class than that of all the other systems that can be observed by them, an assumption which *per se* is just another type of constraint to be applied to the definition of AOIE. One way to avoid this assumption, while still remaining consistent with the fact that AOIE is a stronger form of emergence than is usually considered weak emergence (which generally falls under the ODE) is to classify AOIE as a type of *intermediate emergence* [6]. This kind of terminology has been proposed by Chalmers [6] to deal with a type of emergence that arises from a fundamental epistemological limitation, given the known physical laws at the time of observation. In this sense, intermediate emergence is predicated upon the unbridgeable incompleteness of the observer’s knowledge, so that even ‘in principle’ one would not be able to deduce the macro-level complex behaviour, which is still ‘in principle’ determined by irreducible new laws that one always needs to devise or discover in the future, as also claimed in Cooper [53] where emergence is suggested to be a consequence of uncomputability.

Classifying AOIE as intermediate emergence implies an underlying assumption of the existence (or scientific pertinence) of a stronger form of emergence, which is an open problem. Nevertheless, we consider both hypotheses (i.e. with or without ‘special’ observers) of mathematical and scientific relevance and worth pursuing. For present purposes, since we have only dealt with formal axiomatic theories and not with physical, chemical or biological theories in general, we adhere to what our theoretical results imply, and therefore we avoid the claim of classifying AOIE as either weak, strong or intermediate emergence. What we have shown is that, restricted to the context of AID, FDDDSs, computable systems and formal observer systems, the AOIE is the *strongest* form of emergence that formal axiomatic theories can attain. Algorithmic information and algorithmic randomness have demonstrated and captured fundamental properties that underlie the incompleteness of formal theories and the limits of mathematics [17–19]. Thus, within the scope of this article, it may not come as a surprise that AIT turns out to be the key to formalizing emergence up to the limits that our formal mathematical knowledge can grasp.

## 5. Conclusion

Within the scope of AID, this article studies the fundamental role that algorithmic information plays in the act of observing and in the occurrence of emergent phenomena in discrete deterministic dynamical systems and computable systems.

We have formalized the act of observing a system as mutual perturbations occurring between the observer (which is itself a system) and the observed system. Formal observer systems are systems that already know a formal axiomatic theory, which they can apply in order to compute the future behaviour of the observed system. As a consequence, we show that a (finite discrete deterministic dynamical) system displaying emergent behaviour with respect to an observer constitutes a type of emergence of algorithmic information that is invariant and minimal. Although it depends on the observer’s formal knowledge, this emergence is robust vis-a-vis variations of the arbitrarily chosen method of measuring irreducible information content, errors (or distortions) in the very act of observing and variations of the algorithmic-informational cost of processing the information gathered from the observed system in accordance with the observer’s formal knowledge. Thus, this type of emergence is called ODE.

Then, we investigated the unbounded and rapid increase of emergent algorithmic information, which defines a type of emergence that we call AOIE. In addition to the above invariance, minimality and robustness, any formal axiomatic theory that a formal observer system might devise will eventually fail to compute or predict the behaviour of a system that displays AOIE. Thus, although each formal observer system retains its own subjectivity, as in the above ODE, AOIE defines a type of emergence that outdoes any subjectivity at the asymptotic limit.

We have shown that there is an abstract evolutionary model that displays the temporal (or diachronic) variant of AOIE, which guarantees that no formal observer system is able to always compute the behaviour of evolutionary computable systems in the long run. We have also shown that there is an abstract model for networked systems that displays the holistic variant of AOIE, which guarantees that no formal observer system is able to always compute the expected behaviour of a micro-level subsystem as the size of the macro-level system becomes sufficiently large.

We also compared the ODE and AOIE studied in this article with weak and strong emergence in the literature. Depending on the interpretation of the phrase ‘in principle’ in the usual definitions of weak and strong emergence, AOIE can be classified as weak, intermediate or strong emergence. In any event, the results of the present article show that, within the context of FDDDSs, or computable systems, AOIE is the strongest version of emergence that formal axiomatic theories can grasp or capture. Whether this claim can be extended to stochastic processes, other physical systems and physical theories, and other complexity measures is a problem that needs further discussion and future research. Nevertheless, given the relevance of formal axiomatic theories in mathematics and science in general, we consider the strength of AOIE demonstrated in this article to be remarkable.

## Data Availability

All scripts used in this study are openly accessible through https://github.com/StochasticBiology/boolean-efflux.git. Further information and references to the respective data are provided in the electronic supplementary material [1].

## References

[1] Abrahão FS, Zenil H. 2022 Emergence and algorithmic information dynamics of systems and observers. Figshare. (10.6084/m9.figshare.c.5901204) PMC9125223 35599568

[2] Abrahão FS, Wehmuth K, Ziviani A. 2019 Algorithmic networks: central time to trigger expected emergent open-endedness. Theor. Comput. Sci. **785**, 83-116. (10.1016/j.tcs.2019.03.008)

[3] Adams A, Zenil H, Davies PCW, Walker SI. 2017 Formal definitions of unbounded evolution and innovation reveal universal mechanisms for open-ended evolution in dynamical systems. Sci. Rep. **7**, 997. (10.1038/s41598-017-00810-8) 28428620 PMC5430523

[4] Bedau MA. 1997 Weak emergence. Philos. Perspect. **11**, 375-399.

[5] Hernández-Orozco S, Hernández-Quiroz F, Zenil H. 2018 Undecidability and irreducibility conditions for open-ended evolution and emergence. Artif. Life **24**, 56-70. (10.1162/ARTL_a_00254) 29369710

[6] Chalmers DJ. 2008 Strong and weak emergence. In *The re-emergence of emergence* (eds P Clayton, PCW Davies), Oxford, UK: Oxford University Press. (10.1093/acprof:oso/9780199544318.003.0011).

[7] Fernández N, Maldonado C, Gershenson C. 2014 Information measures of complexity, emergence, self-organization, homeostasis, and autopoiesis. In *Guided self-organization: inception* (ed. M Prokopenko), pp. 19–51. Berlin, Heidelberg: Springer. (10.1007/978-3-642-53734-9_2)

[8] Prokopenko M, Boschetti F, Ryan AJ. 2009 An information-theoretic primer on complexity, self-organization, and emergence. Complexity **15**, 11-28. (10.1002/cplx.20249)

[9] Cover TM, Thomas JA. 2005 Elements of information theory. Hoboken, NJ: John Wiley & Sons, Inc.

[10] Shalizi CR. 2001 *Causal architecture, complexity and self-organization in time series and cellular automata*. PhD Thesis, the University of Wisconsin - Madison.

[11] Lizier J, Bertschinger N, Jost J, Wibral M. 2018 Information decomposition of target effects from multi-source interactions: perspectives on previous, current and future work. Entropy **20**, 307. (10.3390/e20040307) PMC7512824 33265398

[12] Mediano PAM, Rosas F, Carhart-Harris RL, Seth AK, Barrett AB. 2019 *Beyond integrated information: a taxonomy of information dynamics phenomena*. (http://arxiv.org/abs/1909.02297).

[13] Zenil H. 2020 A review of methods for estimating algorithmic complexity: options, challenges, and new directions. Entropy **22**, 612. (10.3390/e22060612) PMC7517143 33286384

[14] Becher V, Figueira S. 2002 An example of a computable absolutely normal number. Theor. Comput. Sci. **270**, 947-958. (10.1016/S0304-3975(01)00170-0)

[15] Zenil H, Kiani NA, Tegnér J. 2017 Low-algorithmic-complexity entropy-deceiving graphs. Phys. Rev. E **96**, 012308. (10.1103/PhysRevE.96.012308) 29347130

[16] Burgin M. 2009 Theory of information: fundamentality, diversity and unification. Singapore: World Scientific.

[17] Calude CS. 2002 Information and randomness: an algorithmmic perspective, 2nd edn. Heidelberg, Germany: Springer-Verlag.

[18] Chaitin G. 2004 Algorithmic information theory, 3rd edn. Cambridge, UK: Cambridge University Press.

[19] Downey RG, Hirschfeldt DR. 2010 Algorithmic randomness and complexity. New York, NY: Springer.

[20] Li M, Vitányi P. 2019 An introduction to kolmogorov complexity and its applications, 4th edn. Cham, Switzerland: Springer.

[21] Wolfram S. 2002 A new kind of science. Champaign, IL: Wolfram Media.

[22] Neary T, Woods D. 2006 P-completeness of Cellular Automaton Rule 110. In *Automata, Languages and Programming* (eds M Bugliesi, B Preneel, V Sassone, I Wegener), pp. 132–143. Berlin, Heidelberg: Springer. (10.1007/11786986_13)

[23] Zenil H, Kiani NA, Marabita F, Deng Y, Elias S, Schmidt A, Ball G, Tegnér J. 2019 An algorithmic information calculus for causal discovery and reprogramming systems. iScience **19**, 1160-1172. (10.1016/j.isci.2019.07.043) 31541920 PMC6831824

[24] Zenil H, Kiani NA, Tegnér J. 2019 The thermodynamics of network coding, and an algorithmic refinement of the principle of maximum entropy. Entropy **21**, 560. (10.3390/e21060560) PMC7515049 33267274

[25] Zenil H, Kiani NA, Zea AA, Tegnér J. 2019 Causal deconvolution by algorithmic generative models. Nat. Mach. Intell. **1**, 58-66. (10.1038/s42256-018-0005-0)

[26] Zenil H, Kiani N, Abrahão F, Tegnér J. 2020 Algorithmic information dynamics. Scholarpedia J. **15**, 53143. (10.4249/scholarpedia.53143)

[27] Zenil H, Kiani NA, Adams A, Abrahão FS, Rueda-Toicen A, Zea AA, Tegnér J. 2022 Minimal algorithmic information loss methods for dimension reduction, feature selection and network sparsification. (http://arxiv.org/abs/1802.05843).

[28] Polani D. 2003 Measuring self-organization via observers. In *Advances in artificial life* (eds W Banzhaf, J Ziegler, T Christaller, P Dittrich, JT Kim), pp. 667–675. Berlin, Heidelberg: Springer. (10.1007/978-3-540-39432-7_72)

[29] Enderton HB. 2001 A mathematical introduction to logic, 2nd edn. Waltham, MA: Academic Press.

[30] Bedau MA. 2010 Weak emergence and context-sensitive reduction. In *Emergence in science and philosophy* (eds A Corradini, T O’Connor). New York, NY and London, UK: Routledge. (10.4324/9780203849408)

[31] Adams AM, Berner A, Davies PCW, Walker SI. 2017 Physical universality, state-dependent dynamical laws and open-ended novelty. Entropy **19**, 461. (10.3390/e19090461)

[32] Davies PCW. 2008 The physics of downward causation. In The *Re-emergence of emergence* (eds P Clayton, PCW Davies). Oxford, UK: Oxford University Press. (10.1093/acprof:oso/9780199544318.003.0002)

[33] Walker SI, Davies PCW. 2012 The algorithmic origins of life. J. R. Soc. Interface **10**, 20120869. (10.1098/rsif.2012.0869) 23235265 PMC3565706

[34] O’Connor T. 2020 Emergent properties. In *The Stanford Encyclopedia of Philosophy* (ed. EN Zalta). Stanford, CA: Metaphysics Research Lab, Stanford University. See https://plato.stanford.edu/archives/fall2020/entries/properties-emergent/.

[35] Bar-Yam Y. 2004 A mathematical theory of strong emergence using multiscale variety. Complexity **9**, 15-24. (10.1002/cplx.20029)

[36] Chaitin G. 2012 Life as evolving software. In *A computable universe: understanding and exploring nature as computation* (ed. H Zenil), pp. 1–23. Singapore: World Scientific Publishing. (10.1142/9789814374309_0015)

[37] Chaitin GJ. 2012 Proving Darwin: making biology mathematical. New York: Pantheon Books.

[38] Chaitin VMFG, Chaitin GJ. 2018 A philosophical perspective on a metatheory of biological evolution. In *The map and the territory: exploring the foundations of science, thought and reality* (eds S Wuppuluri, FA Doria), pp. 513–532. Cham, Switzerland: Springer International Publishing. (10.1007/978-3-319-72478-2_29)

[39] Abrahão FS. 2015 *Metabiologia, Subcomputação e Hipercomputação: em direção a uma teoria geral de evolução de sistemas*. Ph.D. thesis, Federal University of Rio de Janeiro (UFRJ), Brazil, Rio de Janeiro. Language: Portuguese. See http://objdig.ufrj.br/10/teses/832593.pdf.

[40] Abrahão FS. 2016 The ‘paradox’ of computability and a recursive relative version of the Busy Beaver function. In *Information and complexity* (eds C Calude, M Burgin), pp. 3–15. Singapore: World Scientific Publishing. (10.1142/9789813109032_0001)

[41] Hernández-Orozco S, Kiani NA, Zenil H. 2018 Algorithmically probable mutations reproduce aspects of evolution, such as convergence rate, genetic memory and modularity. R. Soc. Open Sci. **5**, 180399. (10.1098/rsos.180399) 30225028 PMC6124114

[42] Abrahão FS, Wehmuth K, Ziviani A. 2018 Emergent open-endedness from contagion of the fittest. Complex Syst. **27**, 369. (10.25088/ComplexSystems.27.4.369)

[43] Barabási A-L. 2016 Network science, 1st edn. New York, NY: Cambridge University Press.

[44] Barabási A-L. 2009 Scale-free networks: a decade and beyond. Science **325**, 412-413. (10.1126/science.1173299) 19628854

[45] Michail O, Spirakis PG. 2018 Elements of the theory of dynamic networks. Commun. ACM **61**, 72-72. (10.1145/3156693)

[46] Abrahão FS. 2016 Emergent algorithmic creativity on networked Turing machines. In *The 8th Int. Workshop on Guided Self-Organization at the Fifteenth Int. Conf. on the Synthesis and Simulation of Living Systems (ALIFE), Cancún*, *Mexico*.

[47] Diestel R. 2017 Graph theory, 5th edn. Heidelberg, Germany: Springer-Verlag.

[48] Lewis TG. 2009 Network science. Hoboken, NJ: John Wiley & Sons, Inc.

[49] Pastor-Satorras R, Vespignani A. 2001 Epidemic spreading in scale-free networks. Phys. Rev. Lett. **86**, 3200-3203. (10.1103/PhysRevLett.86.3200) 11290142

[50] Pastor-Satorras R, Vespignani A. 2002 Immunization of complex networks. Phys. Rev. E **65**, 036104. (10.1103/PhysRevE.65.036104) 11909162

[51] Abrahão FS, Dória FA, Ziviani A. 2020 Learning the undecidable from networked systems. In *Unravelling complexity* (eds S Wuppuluri, FA Doria). Singapore: World Scientific Publishing. (10.1142/9789811200076_0009)

[52] Chaitin GJ. 1975 A theory of program size formally identical to information theory. J. ACM **22**, 329-340. (10.1145/321892.321894)

[53] Cooper SB. 2009 Emergence as a computability-theoretic phenomenon. Appl. Math. Comput. **215**, 1351-1360.

[54] Hodges W. 1993 Model theory, 1st edn. Cambridge, UK: Cambridge University Press.

[55] Lewis H, Papadimitriou CH. 1997 Elements of the theory of computation, 2nd edn. Upper Saddle River, NJ: Prentice-Hall.

[56] Mitchell M. 2009 Complexity: a guided tour. Oxford, UK: Oxford University Press.

[57] Nicolis G, Nicolis C. 2009 Foundations of complex systems. Singapore: World Scientific Publishing Co. Pte. Ltd.

[58] Rich EA. 2007 Automata theory and applications. Upper Saddle River, NJ: Prentice-Hall, Inc.

[59] Rosas FE, Mediano PAM, Jensen HJ, Seth AK, Barrett AB, Carhart-Harris RL, Bor D. 2020 Reconciling emergences: an information-theoretic approach to identify causal emergence in multivariate data. PLoS Comput. Biol. **16**, e1008289. (10.1371/journal.pcbi.1008289) 33347467 PMC7833221

